# Dysfunction of orbitofrontal and dorsolateral prefrontal cortices in children and adolescents with high-functioning pervasive developmental disorders

**DOI:** 10.1186/1744-859X-12-31

**Published:** 2013-10-08

**Authors:** Tetsuji Sawa, Masaki Kodaira, Arata Oiji, Daimei Sasayama, Yoshitaka Iwadare, Hirokage Ushijima, Masahide Usami, Kyota Watanabe, Kazuhiko Saito

**Affiliations:** 1 Department of Developmental Psychiatry, Kitasato University Graduate School of Medical Science, 1-15-1 Kitasato, Minami-Ku, Sagamihara, Kanagawa 252-0374, Japan; 2 Department of Child and Adolescent Mental Health, Aiiku Hospital, 5-6-8 Minamiazabu, Minato-Ku, Tokyo 106-8580, Japan; 3 Department of Neuropsychiatry, Shinshu University School of Medicine, 3-1-1 Asahi, Matsumoto, Nagano 390-8621, Japan; 4 Department of Child and Adolescent Psychiatry, National Center for Global Health and Medicine, Kohnodai Hospital, 1-7-1 Kohnodai, Ichikawa, Chiba 272-0836, Japan

**Keywords:** Pervasive developmental disorders, Childhood, Adolescence, Iowa gambling task, Wisconsin card sorting test, Orbitofrontal cortex, Dorsolateral prefrontal cortex

## Abstract

**Background:**

Several lines of evidence suggest that dysfunction of the dorsolateral prefrontal cortex (DLPFC) and orbitofrontal cortex (OFC) contributes to the pathophysiology of pervasive developmental disorders (PDD). The purpose of this study was to investigate neuropsychological dysfunctions in both the DLPFC and OFC of children and adolescents with high-functioning PDD.

**Methods:**

The Iowa gambling task (IGT), which reflects OFC function, and the Wisconsin Card Sorting Test (WCST), which reflects DLPFC function, were assigned to 19 children and early adolescents with high-functioning PDD and 19 healthy controls matched for gender, age, and intelligence.

**Results:**

Compared to healthy controls, patients with high-functioning PDD displayed poorer performance on the IGT and the WCST.

**Conclusions:**

These results indicate that both the DLPFC and OFC could be impaired in children and early adolescents with high-functioning PDD.

## Background

Pervasive developmental disorders (PDDs) are a group of mental disorders that are characterized by multiple developmental delays in several basic functions such as cognition, socialization, and communication. PDDs include autistic disorder, Asperger's disorder, Rett's disorder, childhood disintegrative disorder, and pervasive development disorder not otherwise specified (PDD-NOS) by the Diagnostic and Statistical Manual of Mental Disorders (DSM-IV-TR). In the DSM-5 classification, the diagnosis of autistic disorder spectrum disorder encompasses the previous DSM-IV-TR diagnoses of autistic disorder, Asperger's disorder, childhood disintegrative disorder, and PDD-NOS. Autistic disorder is characterized by severe and pervasive impairment of several developmental functions, such as reciprocal social interaction skills, verbal and nonverbal communication, and the presence of stereotyped behavior, interests, and activities. Asperger's syndrome is similar to autistic disorder in that it is characterized by social and verbal impairments but differs in that it shows relatively normal language and cognitive development.

The etiology of PDD is hitherto unknown, but brain dysfunctions have been implicated in children and adults with PDD. Many studies have investigated whether functions of various brain regions are altered in PDD. However, none indicates a unified view on the specific regions that are affected in PDD. Considering that the core symptoms of PDD include impaired communication and social skills and that the frontal lobes are known to participate in these functions, it is likely that frontal lobe dysfunctions play a major role in PDD.

Several neuropsychological studies have revealed impaired executive functions in PDD patients. Executive functions are a set of mental processes that controls goal-directed behavior, which includes planning, working memory, attention, problem solving, verbal reasoning, inhibition, mental flexibility, task switching, and initiation and monitoring of actions. The Wisconsin Card Sorting Test (WCST) is among the most frequently administered neuropsychological tests that assess executive functions. Positron emission tomography (PET) studies have indicated that WCST performance is associated with activity in the dorsolateral prefrontal cortex (DLPFC) [1]. PDD patients, particularly those with autistic disorder, tend to perform worse on the WCST than healthy subjects by committing a significantly higher number of perseverative errors and achieving fewer categories [2-4].

Recently, altered function of the orbitofrontal cortex (OFC) has been implicated in the neuropsychological pathophysiology of PDD, in the context that PDD patients exhibit impaired social behavior, social interaction, and attention. Bachevalier [5] hypothesized that abnormalities in the orbitofrontal-amygdala circuit may be a fundamental underlying mechanism in PDD. The OFC, especially its right lateral subdivision, appears to play an important role in social cognition [6,7] and in the pathophysiology of autistic disorder [5,8,9]. Performance on the Iowa gambling task (IGT), which evaluates real-life decision-making abilities under ambiguous conditions, is particularly sensitive to changes in OFC function [10,11]. Nevertheless, only a few studies have used the IGT to examine individuals with PDD.

In the present study, we hypothesized that children and adolescents with high-functioning PDD have lower DLPFC and OFC functions than healthy individuals. To test this hypothesis, we assigned the WCST and IGT to children and adolescents with high-functioning PDD and compared their performance on these tests with that of gender-, age-, and intelligence-matched healthy subjects.

## Methods

### Subjects

Study participants included 19 Japanese patients with autistic disorder or Asperger's disorder (aged 10–15 years) who were admitted as either inpatients or outpatients at Kohnodai Hospital, National Center for Global Health and Medicine. In order to include only patients with overt clinical features of PDD, those diagnosed as PDD-NOS were not included in the study. Two trained child and adolescent psychiatrists diagnosed their condition according to the DSM-IV-TR criteria [12]. Patients with a current or past diagnosis of mood disorders, schizophrenia, substance-related disorders, attention-deficit and disruptive behavior disorders, or mental retardation were excluded from this study. Eleven patients (ten males and one female) were diagnosed with autistic disorder, and eight patients (seven males and one female) were diagnosed with Asperger's syndrome. Normal healthy controls were recruited from two local public schools by word of mouth, and a total of 47 students volunteered to participate. A child psychiatrist conducted a 30-min clinical interview with each potential control subject to rule out any prevalent or past history of psychiatric disorders. Subjects with prevalent or past history of psychiatric diagnosis, those who underwent treatment for psychiatric or psychological disorders, or those who had a history of absenteeism from school were excluded from the control group. Two of the 47 volunteers were excluded because they exhibited obsessive symptoms: one because of tic disorder, and three because of PDD. After matching these subjects with the patients for gender, age, handedness, and intelligence, 19 volunteers were enrolled as control subjects.

### Assessment

Handedness was determined for each subject using the hand usage questionnaire [13]. The IGT [14] was assigned by a trained child and adolescent psychiatrist following the previously described procedures of the original IGT version. The only difference from the original task was that play money was converted from US dollars to Japanese yen [15]. The IGT requires subjects to repeatedly select 100 cards from four decks and maximize their profit. The goal is to win as much money as possible, or, as far as possible, avoid losing money. To achieve this, subjects must discover the most advantageous decks and preferentially select cards from them. Each time they turn over a card, subjects win money; however, when turning over a card, subjects sometimes have to pay a penalty according to a preprogrammed reward/punishment schedule. Gains and losses are different for each card selected from the four decks. Although they yield 10,000 yen rewards, decks A and B are 'disadvantageous’ because their penalties are higher than those of decks C and D. Therefore, they cost more in the long run. These decks are also termed high-paying decks. Decks C and D are 'advantageous’ because while they yield only 5,000 yen, their penalties are lower in amount to gains in the long term. These decks are also termed low-paying decks. Thus, successful task performance requires frequent sampling from decks C and D than from decks A and B. In the present study, subjects were informed that they would get a special gift if they earned at least 300,000 yen at the end of the task [16]. The 100 selections were divided into five blocks of 20 selections each, which allowed us to verify changes in selection patterns throughout the experiment. We recorded the number of disadvantageous cards selected in the overall task and in each block of 20 selections. Four clinical psychologists individually administered the Wechsler Intelligence Scale for Children-Third Edition and the Keio version of the WCST [17]. The WCST was used to determine executive function or abstract reasoning that involves working memory [1] and to examine DLPFC function [18]. In the WCST, we evaluated the number of categories achieved (CA), total errors (TE), and perseverative errors (PE) as reported by Nelson [19]. Categories achieved indicated the ability to change abstract categories, and PEs represent perseveration of preceding errors or the inability to inhibit preceding incorrect responses.

### Statistical analyses

Data were analyzed using the Statistical Package for the Social Sciences (SPSS; IBM, Armonk, NY, USA) version 16 for Windows (Microsoft, Redmond, WA, USA). We compared continuous variables (i.e., age and IQ) using *t* tests and ordinal variables using the Mann-Whitney test. The results were considered statistically significant at *p* < 0.05.

### Ethical considerations

This study was designed in accordance with the Declaration of Helsinki and approved by the Ethics Committee of the National Center for Global Health and Medicine. Written informed consent was obtained from all participants and their parents after they had received a description of the study.

## Results

**Figure 1:**
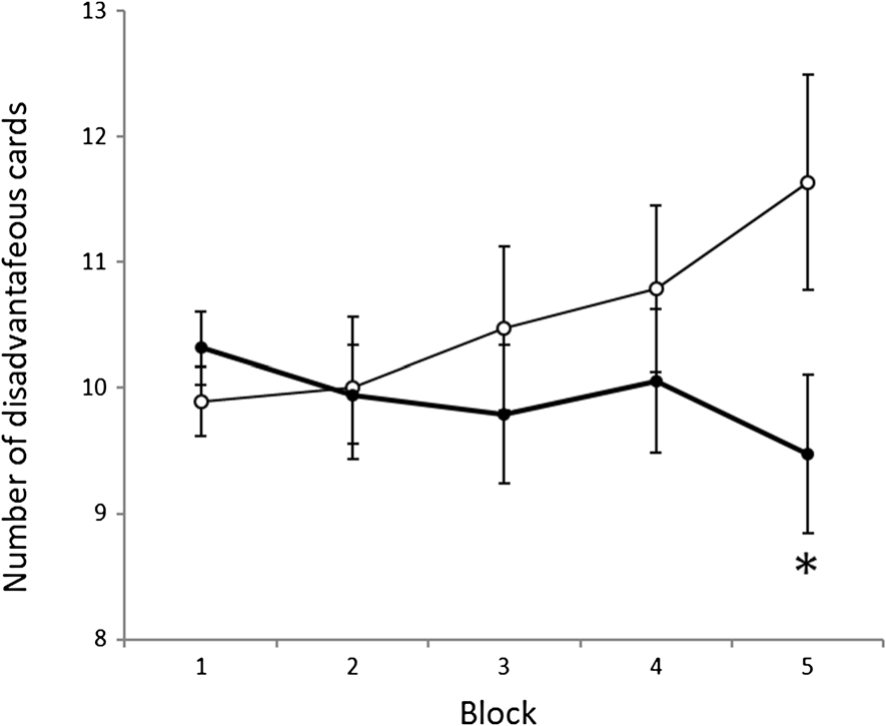
**Order of card selection in the Iowa gambling task from the first to the fifth selection.** The *solid* and *dotted lines* show the mean number of disadvantageous cards drawn by PDD patients and controls, respectively, in each selection of 20 cards. Horizontal bars indicate the standard error of the means. Children with PDD chose more cards from the disadvantageous deck towards the end of the task. *P < 0.05 (Mann-Whitney test, significantly greater than controls).

Demographic and clinical characteristics of the study participants are summarized in Table 1. PDD patients had not been taking medications at the time of the study. The following comorbidities were observed in the patients: stereotypic movement disorder (three patients), tic disorders (two patients), trichotillomania (one patient), selective mutism (one patient), and specific phobias (one patient). In the WCST, PDD patients achieved significantly fewer categories (CA) and committed significantly more TEs and PEs than the controls (Table 2). During the last selection of the IGT (selections 81–100), the number of disadvantageous card selected was significantly higher in PDD patients than in controls (Figure 1). During the other selections in IGT, we observed no significant differences between PDD patients and the controls.

**Table 1:** Subject characteristics

**Characteristic**	**PDD (*n *= 19)**	**Controls (*n *= 19)**	***P *value**
Age (months) (mean ± SD)	158.84 ± 21.48	157.42 ± 23.81	0.848
Full IQ (mean ± SD)	95.95 ± 12.83	97.32 ± 9.48	0.711
Verbal IQ (mean ± SD)	92.96 ± 13.54	97.21 ± 12.88	0.652
Performance IQ (mean ± SD)	95.26 ± 13.54	97.84 ± 7.24	0.880
Gender (*n* (%))			
Male	17(89.5)	17(89.5)	
Female	2(10.5)	2(10.5)	
Handedness (*n* (%))			
Right-handed	16(84.2)	16(84.2)	
Left-handed	2(10.5)	2(10.5)	
Ambidextrous	1(5.3)	1(5.3)	

The demographic and clinical characteristics of the study subjects are shown. *PDD* pervasive developmental disorders, *SD* standard deviation, *IQ* intelligence quotient.

**Table 2:** Subject performance on the Wisconsin card scoring test

	**PDD (*n *= 19)**	**Controls (*n *= 19)**	***P *value**
	**Mean ± SD**	**Mean ± SD**	
CA	2.53 ± 1.96	4.21 ± 1.44	0.004
TE	23.58 ± 10.47	15.79 ± 8.19	0.015
PE	7.89 ± 4.52	3.58 ± 4.75	0.025

Subject performances on the Wisconsin Card Scoring Test are shown. *CA* categories achieved, *TE* total errors, *PE* perseverative errors.

## Discussion

In the present study, PDD patients committed significantly more perseverative errors and achieved fewer categories in the WCST than the controls. Our results are consistent with those of previous studies in which WCST was assigned to individuals with high-functioning autistic disorder in late adolescence and early adulthood [3,4]. Patients with DLPFC damage also show more perseverative errors in the WCST than controls [20], and DLPFC activation measured by PET [21] and functional magnetic resonance (fMRI) [22] is increased in subjects performing the WCST. Proton magnetic resonance spectroscopy has revealed similar dysfunction in the left DLPFC and anterior cingulate cortex in children with autistic disorder [23]. Courchesne et al. [24] reported that the mean number and size of DLPFC and medial prefrontal cortex neurons are higher than those of control subjects. Thus, the results of the WCST in the present study are consistent with the findings of previous studies employing anatomical or neuroimaging techniques.

In the present study, PDD patients showed a significantly stronger tendency toward selecting disadvantageous cards in the IGT than the controls, even during the late examination period. To our knowledge, only one other study has investigated the results of IGT assigned to children and adolescents with PDD, which showed no significant difference between patients with Asperger's syndrome and controls [25]. This study showed that the percentage for selecting disadvantageous cards in the final selection in the control group was 31.6%, whereas that of the Asperger's syndrome group was 43.6%. We suspect that the nonsignificant results could be because of a smaller sample size of patients or the low severity of PDD that they exhibited. Future studies with larger sample sizes are needed to elucidate this relationship. Other studies have reported that OFC-injured patients also show a tendency to select disadvantage cards continuously in the later selections [10,11].

Using morphometric MRI, Hardan et al. [26] observed that the total volume (i.e., gray plus white matter) in the right lateral OFC was decreased in children and adolescents with autistic disorder but was increased in adults with autistic disorder. Moreover, a recent fMRI study of healthy adults reported that the processing theory of mind tasks, a function believed to be impaired in individuals with autistic disorder, was associated with increased activation of the right lateral OFC [7]. The results of the IGT in this study seem to fit with the findings of these previous neuroimaging studies. Thus, considering the results of this study and those of previous studies, PDD patients may have some structural and functional abnormalities in the OFC. Furthermore, the results of this study indicate that both DLPFC and OFC functions are impaired in high-functioning PDD patients in childhood and early adolescence.

The limitations of this study are as follows: (1) the sample size was small, and the PDD subjects were recruited from only one facility; (2) because of a narrow age range and a smaller number of female patients, we could not examine any effects of sex/age differences; (3) only neuropsychological tests were employed in this study. These neuropsychological tests cannot verify frontal lobe dysfunctions directly.

## Conclusions

We assigned the WCST and IGT to high-functioning PDD patients in childhood and early adolescence with controls matched for gender, age, handedness, and intelligence. We identified that both DLPFC and OFC functions are impaired in PDD patients. This study is the first to examine the impairment of both DLPFC and OFC functions in children and adolescents with high-functioning PDD. The findings of this study will contribute toward the understanding of brain dysfunction and clinical features of PDD.

To achieve this end, joint studies involving multiple facilities with larger sample sizes and wider age ranges need to be conducted. Moreover, studies evaluating the relationships between frontal lobe functions, and mind tasks and social skills also need to be conducted.

## Competing interests

The authors declare that they have no competing interests.

## Authors’ contributions

TS wrote the manuscript. MK and AO designed the study. MK, YI, HU, MU, and KW recruited and screened the study participants. MK and YI diagnosed the study participants. MK administered the IGT. TS undertook the statistical analysis. AO and KS supervised the data analysis and manuscript writing. DS gave critical comments on the manuscript. All authors have contributed to and approved the final manuscript.
